# Evaluation of cytotoxic, genotoxic and inflammatory responses of nanoparticles from photocopiers in three human cell lines

**DOI:** 10.1186/1743-8977-10-42

**Published:** 2013-08-22

**Authors:** Madhu Khatri, Dhimiter Bello, Anoop K Pal, Joel M Cohen, Susan Woskie, Thomas Gassert, Jiaqi Lan, April Z Gu, Philip Demokritou, Peter Gaines

**Affiliations:** 1 Department of Work Environment, One University Avenue, University of Massachusetts Lowell, Lowell, MA 0185, USA; 2 Department of Biological Sciences, One University Avenue, University of Massachusetts Lowell, Lowell, MA 0185, USA; 3 Biomedical Engineering and Biotechnology Program, One University Avenue, University of Massachusetts Lowell, Lowell, MA 0185, USA; 4 University of Massachusetts Worcester, 55 Lake Avenue North, Worcester, Massachusetts 01655, USA; 5 Department of Environmental Health, Harvard School of Public Health, Boston, MA, USA; 6 Civil and Environmental Engineering Department, Northeastern University, Boston, MA, USA

**Keywords:** Engineered nanoparticles, Photocopier, Printer, Toner, Inflammation, Apoptosis, DNA damage

## Abstract

**Background:**

Photocopiers emit nanoparticles with complex chemical composition. Short-term exposures to modest nanoparticle concentrations triggered upper airway inflammation and oxidative stress in healthy human volunteers in a recent study. To further understand the toxicological properties of copier-emitted nanoparticles, we studied *in-vitro* their ability to induce cytotoxicity, pro-inflammatory cytokine release, DNA damage, and apoptosis in relevant human cell lines.

**Methods:**

Three cell types were used: THP-1, primary human nasal- and small airway epithelial cells. Following collection in a large volume photocopy center, nanoparticles were extracted, dispersed and characterized in the cell culture medium. Cells were doped at 30, 100 and 300 μg/mL administered doses for up to 24 hrs. Estimated dose delivered to cells, was ~10% and 22% of the administered dose at 6 and 24 hrs, respectively. Gene expression analysis of key biomarkers was performed using real time quantitative PCR (RT-qPCR) in THP-1 cells at 5 μg nanoparticles/mL for 6-hr exposure for confirmation purposes.

**Results:**

Multiple cytokines, GM-CSF, IL-1β, IL-6, IL-8, IFNγ, MCP-1, TNF-α and VEGF, were significantly elevated in THP-1 cells in a dose-dependent manner. Gene expression analysis confirmed up-regulation of the TNF-α gene in THP-1 cells, consistent with cytokine findings. In both primary epithelial cells, cytokines IL-8, VEGF, EGF, IL-1α, TNF-α, IL-6 and GM-CSF were significantly elevated. Apoptosis was induced in all cell lines in a dose-dependent manner, consistent with the significant up-regulation of key apoptosis-regulating genes P53 and Casp8 in THP-1 cells. No significant DNA damage was found at any concentration with the comet assay. Up-regulation of key DNA damage and repair genes, Ku70 and Rad51, were also observed in THP-1 cells, albeit not statistically significant. Significant up-regulation of the key gene HO1 for oxidative stress, implicates oxidative stress induced by nanoparticles.

**Conclusions:**

Copier-emitted nanoparticles induced the release of pro-inflammatory cytokines, apoptosis and modest cytotoxicity but no DNA damage in all three-human cell lines. Taken together with gene expression data in THP-1 cells, we conclude that these nanoparticles are directly responsible for inflammation observed in human volunteers. Further toxicological evaluations of these nanoparticles, including across different toner formulations, are warranted.

## Introduction

Photocopiers and printers are commonly used electronic devices in offices, industrial settings, and households. These devices are a potential source of indoor air pollutants as they emit nanoparticles (NP), and other gaseous pollutants [1-4]. Past research has focused on emission of volatile organic compounds (VOCs), ozone, and other gaseous pollutants, results of which prompted improved technologies that reduced exposures to such pollutants (e.g. ozone). More recently, the focus has shifted to the investigation of nanoparticle emissions, their origin and chemical composition [5]. The peak size distribution of emitted nanoparticles is often below 50 nm, whereas the toner particles are several microns large (commonly 5–20 μm) [4-8]. Nanoparticles emitted during photocopier operation are formed during the image transfer process.

The chemical composition of nanoparticles emitted from photocopiers is best described as a mixture of organic compounds and inorganic metal oxide additives and reflects the complex toner chemistry, as shown recently for one type of photocopier [5] and select printers [8]. The organic fraction of these nanoparticles is formed primarily from the condensation of semi-volatile organic compounds (SVOCs) evaporated from the toner and possibly other paper constituents during the printing/photocopying process, and remains poorly characterized [2,7]. The inorganic fraction of airborne NP varies with the toner formulation and may contain variable amounts of silicon (Si), sulphur (S), titanium (Ti), iron (Fe), chromium (Cr), nickel (Ni), zinc (Zn) and possibly other elements, which were also found in toners. They more likely originate from metal oxide additives in toners (e.g. fumed silica, titania, magnetite), associated impurities, and possibly the paper.

The toxicology of nanoparticles emitted from photocopiers and printers is poorly understood, in part because this issue has received little attention. Theegarten et al. [9] describes an interesting case study in which peritoneal biopsies of a worker exposed to NP emitted from printer revealed large deposits of black material comprised of carbon-based aggregates of nanoparticles in the submesothelial tissues. The authors hypothesized that inhaled NP translocated to the submesothelial tissue via lymphatic and blood vessels. Several studies over the past decade have reported elevated markers of genotoxicity in the peripheral blood of copy center workers. For example, Balakrishnan and Das [10] and Gadhia et al. [11] reported significant chromosomal aberration in photocopier workers as compared to controls. Other studies reported significant DNA damage and chromosomal aberrations in the peripheral blood samples of workers occupationally involved in photocopying [12,13]. Similarly, Manikantan et al. reported significantly higher DNA damage compared to controls in a different demographic population of photocopier workers [14]. Of note, aforementioned human studies lacked quantitative exposure assessment, had poor exposure histories of the individuals studied, and did not pinpoint the actual exposure triggers leading to the observed genotoxic effects.

More recently, Tang et al. investigated cytotoxicity and genotoxicity of laser printer emissions in human A549 lung cells using an air-to-liquid delivery system [15]. They found that emissions of two out of five printers were genotoxic. Gminski et al. [16] demonstrated genotoxic effects of the organic extracts (in dimethyl sulfoxide) of three commercially available bulk toner materials in cultured human epithelial A549 lung cells. It should be noted, however, that the chemistry and sizes of particles emitted by copiers may be substantially different from the bulk toner material or its organic solvent extracts. Therefore, the toxicological outcomes from studies that use bulk toner material instead of the emitted airborne NPs may differ significantly.

In a recent study we have shown that single, short-term (6 h) exposures in a photocopy center environment at modest exposure levels (daily averages of 5,000-30,000 particles/cm^3^) induced statistically significant increases in systemic oxidative stress (measured as *8-OH-dG* in urine) and upper airway inflammation (2–10 fold as indicated by recruitment of neutrophils, increased cytokines expression and total proteins in nasal lavages) when post-exposure levels were compared to background levels for healthy controls [17]. In contrast to prior studies, several instruments were used in this study to monitor in real time airborne nanoparticle concentrations, ozone levels, VOCs, and other indoor air quality parameters (CO_2_, CO, relative humidity, temperature) of the exposed healthy volunteers. Airborne nanoparticle concentration was the only measured exposure parameter that differed significantly between exposure and background environments. While classification of individuals based on nanoparticle exposure status (exposed vs. non-exposed controls) resulted in clear exposure-response relationships, the quantitative relationships between inflammatory biomarkers and average particle number concentration was less clear, raising the possibility that other trace organic pollutants (e.g. formaldehyde, etc.) may also be involved. This finding and the lack of prior toxicological data on these nanoparticles made it necessary to also study *in-vitro* their toxicological properties.

In the present study, we investigated the cytotoxic effects on human cell lines of NPs collected from the same photocopy center that participated in the human study in order to better understand whether and how the process of inflammation observed in human volunteers could be induced by exposure to airborne nanoparticles. It has been proposed that oxidative damage and inflammation are key mechanisms responsible for adverse health effects of particulate matter in general, including nanoparticles [18,19]. Hence, in this initial investigation we focused specifically on NP-induced cytokine production, DNA damage, and apoptosis, to match them with the endpoints measured in the human volunteers study [17]. We chose three cell types, specifically human macrophages [phorbol 12- myristate 13-acetate (PMA) - differentiated THP-1 cells], primary human nasal epithelial cells and primary human small airway epithelial cells, because the respiratory epithelium and macrophages are the cells that come in direct contact with inhaled nanoparticles and are therefore reasonable *in-vitro* surrogates for studying nanoparticle toxicity.

## Materials and methods

### Collection of airborne nanoparticles

The photocopy center, its activities, and exposure characterization studies are described in more detail in Bello et al. [5]. The center used two large volume copiers from a major manufacturer. Airborne nanoparticles were collected using the Harvard Compact Cascade Impactor (Harvard CCI) [20], a high volume (30 L/min) size selective particle sampler, described in [5], which enables the collection of large quantities of size- fractionated particles for physicochemical and toxicological characterization studies. The nanosize fraction was collected on a Teflon filter, whereas the fine particulate matter (PM_0.1–1.0_) and coarse fractions (PM_2.5–10_) were collected in pre-cleaned polyurethane foams. The sampler was located next to the exhaust port of one of the copy machines, one of the locations with the highest nanoparticle emissions. Sampling was conducted only during the work hours (7 AM-3 PM). In order to minimize collection of incidental nanoparticles in background air, the sampler was turned off when the copy machines were idle. Collection of sufficient mass of the nanosized fraction required continuous sampling for two-three weeks per sample during the Winter of 2010 and Spring of 2011, a period of maximum photocopying activity in the center. A total of three samples were collected for this study. The particulate mass collected on each fraction (including the nano-sized fraction) was determined gravimetrically as the difference between the post- and pre-weight of filters. The filter weight was determined in a temperature and humidity controlled chamber following at least 24 hrs equilibration time, using a Mettler-Toledo (Model-XP 26) microbalance. The mass of the nanoscale fraction (on Teflon filters) was in the range 150–300 μg/sample.

A suite of real-time instruments, including a fast mobility particle sizer (Model 3091, TSI Inc.), an aerodynamic particle sizer (Model APS 3021, TSI Inc.) and a condensation particle counter (CPC 3007, TSI Inc.), were also used to check the size distributions and total particle number concentration during the sampling days and compare them with background values. Size distributions were found to be fairly stable. Average background total particle number concentration at the beginning of the workday was in the range of 2000–3000 particles/cm^3^, whereas at the sampler location it was > 30,000/cm^3^ and peak maxima of over one million particle/cm^3^ were recorded. Based on the real time number concentration data, we estimated the overall mass contribution from the background nanoparticles at ~6%.

### Physicochemical and morphological characterization of emitted nanoparticles

**Figure 1:**
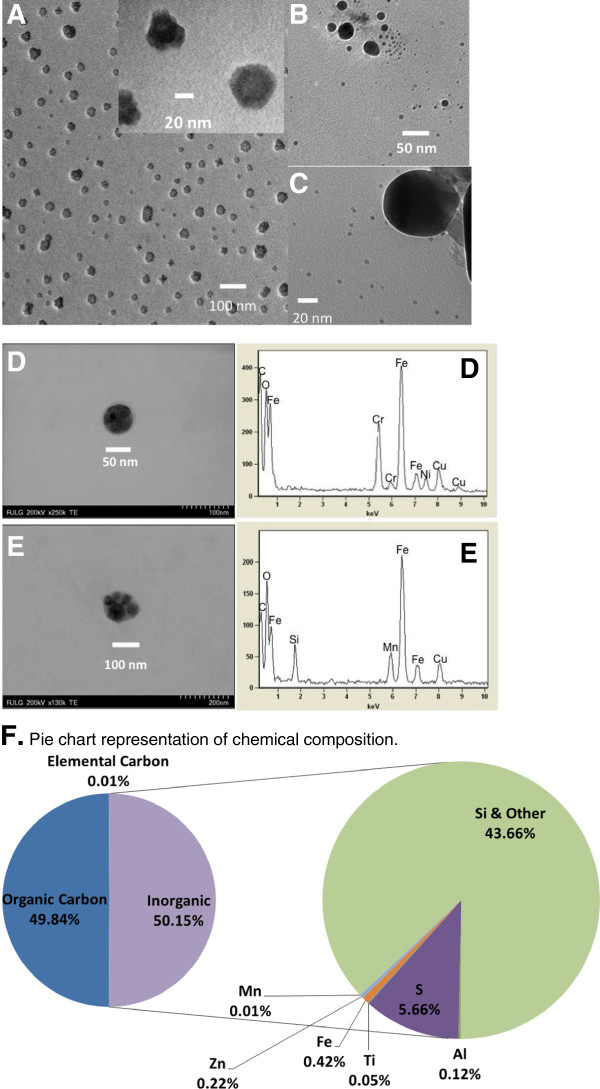
**Representative TEM images of airborne nanoparticles. (A) (B)** and **(C)** illustrate the most commonly observed morphologies of airborne nanoparticles emitted from the photocopy machines, A smaller number of particles contained heavy metals, as illustrated by the energy dispersive spectroscopy spectra in images **(D)** and **(E)**. **F**, Pie chart representation of chemical composition. [Cu signal is from the TEM grid]. (Reproduced with permission from Bello et al 2012).

Extensive physicochemical and morphological characterization of airborne nanoparticles in support of the subsequent toxicological evaluation work was performed, and the data is presented in a separate publication [5]. Briefly, the chemical composition of the collected NPs was complex and reflected the complex chemistry of the toner formulation. The airborne nanoscale fraction contained ~50% organic carbon and 0.1% elemental carbon, with the remainder being of inorganic origin. The most abundant elements in the inorganic fraction were sulfur (S, 5.7%), silicon (Si, 0.6%) and iron (Fe, 0.42%), as well as smaller amounts (1% or less) of zinc (Zn, 0.22%), aluminum (Al, 0.12%), titanium (Ti, (0.05%), tin (Sn, 0.01%), manganese (Mn, 100 ppm), phosphorus (P, 560 ppm), and magnesium (Mg, 650 ppm), all of these elements were also present in the bulk toner formulation and dust collected from the exhaust port of the photocopier. The water-soluble fraction varied with the element from ~8-9% for Fe and Al to >90% for Ti, Zn and S [5]. Several long chain alkanes (C24, or tetracosane to C40, tetracontane) were found in the nanoscale fraction in the same relative abundance as in bulk toners, the most abundant being tetracontane, octatriacontane, and hexatriacontane. The nanoscale fraction contained only traces (10–50 parts per billion, ppb) of a few species of polycyclic aromatic hydrocarbons (phenanthrene, fluoranthene, pyrene, chrysene, and benzo(b)fluoranthene), not present in the toner. Table 1 and Figure 1 summarize the metal content in the nanoscale fraction and representative morphology and elemental analysis of nanoparticles, respectively.

**Table 1:** Magnetic sector inductively coupled plasma mass spectrometry (SF-ICP-MS) analysis of the nanoscale fraction collected with the Harvard Compact Cascade Impactor (CCI)

**Element**	**Fe**	**Ti**	**Si **^**a**^	**Mn**	**Al**	**Zn**	**S**	**Cu**	**Cr**	**Ni**	**Mo**	**Mg**	**Ca**	**P**
**Total**														
**(SD)**	4393	5671	5711	99.3	1371	2312	59087	286.3	39.7	101.1	38.4	679.0	2609	559.6
**(μg/g)**	(219)	(254)	(2690)	(5.8)	(69.4)	(122)	(2803)	(17.1)	(2.6)	(5.6)	(1.73)	(36.9)	(254)	(45.6)
**Water soluble**														
**(SD)**	395.4	0.78	n/a	48.81	111.1	2263	60196	128.5	8.12	51.21	21.84	395.4	2421	553.3
**(μg/g)**	(11.7)	(0.53)		(1.48)	(2.7)	(28.1)	(262)	(2.54)	(0.32)	(2.92)	(0.16)	(11.73)	(104.71)	(61.8)
**% Water soluble**	9	93	n/a	49	8.1	98	101	45	20	51	57	58	93	99

Elemental analysis targeted 47 elements,of which the following were the most abundant. Other elements of toxicological relevance (V, Co, As, Cd, Pb) were detected in trace amounts. The remainder elements tested for were: Li, B, K, Sc, Rb, Sr, Y, Nb, Rh, Pd, Ag, Sn, Sb, Cs, Ba, La, Ce, Pr, Nd, Sm, Eu, Dy, Ho, Yb, Lu, Pt, Th, U. Values are rounded to the nearest integer.

^a^ Silicon is an estimate based on reanalysis of initial digests and it is likely to be underestimated in some fractions. Values have been corrected for recovery.

n/a, not analyzed.

### Extraction of nanoparticles, dispersion, and characterization

The Teflon filters were submerged in 5 mL of high purity water in a precleaned closed-lid scintillation vial and sonicated in a water bath for 1 hr to detach the particles from the filter, followed by cup sonication Branson Sonifier S-450A (Branson Ultrasonics, Danbury, CT) for 1 min at 30% amplitude (maximum power output of 400 Watts at 60 Hz), with 10 sec cycle to deagglomerate the particles. The solution was concentrated to ~1 mL using a Rotavapor R-215 (BUCHI corporation, Newcastle, DE) at room temperature. The recovery efficiency of particles from the filter calculated from gravimetric measurements (mass gain on extraction vial/nanoparticle mass on filter) and was found to be 92%.

Stable particle dispersions were then prepared using previously described standardized protocols [20], which included the calibration of sonication equipment, standardized reporting of sonication energy, and chaacterization of the critical delivered sonication energy (DSEcr). In brief, nanoparticle dispersions were sonicated in DI water with a calibrated Branson Sonifier S-450A (Branson Ultrasonics, Danbury, CT), according to a published calorimetric calibration method [20,21]. The power delivered to the sample was 1.75 Watts. Dispersions were then analyzed for hydrodynamic diameter (d_H_), polydispersity index (PdI), and zeta potential (ζ) by dynamic light scattering (DLS) using the Zetasizer Nano-ZS (Malvern Instruments, Worcestershire, UK). Plots of the hydrodynamic diameter as a function of DSE exhibiting asymptotic deagglomeration trends were derived for each ENM to determine the critical DSE necessary to achieve stable monodisperse suspensions (data not shown). Each sample was then dispersed at this experimentally determined DSE_cr_, which for copier emitted and reference nanoparticles was in the range of ~ 160 to 240 J/mL, and required sonication for 15–20 min (data not shown).

A stock NP solution of 0.5 mg/mL was prepared in the cell culture media (cell type dependent), from the previous NP dispersions in DI water. This NP stock in culture medium was further used to dose the cells at final NP concentrations of 30, 100 and 300 μg/mL. The 0.5 mg/mL stock solution of NP suspensions was incubated with 10 μg/mL Polymyxin B (VWR, Bellefonte, PA Cat # 80058–572) for 60 min to make it endotoxin-free before cell dosing.

The effective density of particle agglomerates in liquid suspension was measured following a method of volumetric centrifugation recently developed by the authors [21]. The temperature of the laboratory was kept at 22 ± 2.5°C during all size measurement experiments. The average hydrodynamic size of nanoparticles in dispersion from triplicate readings was 156.2 nm (±1.0) with a PDI of 0.34 (± 0.015) and the measured effective density for agglomerates in suspension was 1.06 g/cm^3^. Zeta potential values, a measure of surface charge, were strongly negative for suspensions in DI H_2_O (mean ± SD, ~ −35 mV ±4.0), and less negative in cell culture medium (~ −10 mV ± 1.0). The dispersions were stable for at least 48 hours.

### Control nanoparticles

Copper oxide nanoparticles, with primary particle size of d_BET_ 58 nm and specific surface area 17.2 m^2^/g (Sigma Aldrich, St Luis, MO) were included as positive reference nanoparticles as they are well-known to be toxic under *in-vitro* conditions [22]. The d_H_ and PdI of CuO dispersions in cell media, following previously described protocols, was as follows: RPMI (d_H_ 268.8 nm ± 5.3, PdI 0.21); nasal (301.3 nm ± 10.1, 0.27), small airways (215.7 nm ± 3.8, 0.14), and DI water (310 nm ± 7.6, 0.32). Phosphate buffer saline (PBS, VWR, Bellefonte, PA) and poly lactic-co-glycolic acid (PLGA) polymeric beads (180 nm; generous gift from Dr. Suresh Gadde at Brigham and Women’s hospital, MA) were included as negative control for each time point in all the treatment cell culture plates. Supernatants collected from cells induced with lipopolysaccharide (LPS, Sigma, St. Lois, MO) (1 μg/mL final concentration) were used as reference positive control for cytokine analysis. As mentioned earlier, nanoparticles were treated with Polymyxin B to neutralize any endotoxin contamination. In order to explore any potential interactions of nanoparticle with endotoxins (as it may happen in the real world), another dose of nanoparticles (300 μg/mL) not treated with Polymyxin B was also used and compared with the same dose treated with Polymyxin B.

For Annexin V experiments, carbon black N550 (CB, Cabot Corp. Billerica, MA), 39.2 m^2^/g, 44 nm primary particle size, was also included [23]. The reason was to assess possible interferences with the detection system, especially given that the collected nanoparticle fraction was black in color and we initially suspected that some of the 5-10% carbon black in the toners could become airborne. Subsequent chemical analysis confirmed absence of significant amounts of carbon black in the airborne nanoscale fraction, and its use was discontinued from further comparative assessment.

### Cell culture

The human monocytic immortalized cells THP-1 (American Type Culture Collection, USA), were cultured in RPMI 1640, 2 mM L-glutamine supplemented with 10% FBS. The primary human nasal epithelial cells and small airway epithelial cells (Promo cell, Germany) were cultured using the commercially available Airway Epithelial Cell Growth Medium and Small Airway Epithelial Cell Growth Medium (Catalog number C 21060 and C21070, Promo cell, Germany) without additional FBS supplementation. All the media were supplemented with 1% penicillin-streptomycin and amphotericin. The generic cell culture protocol consisted of growing cells in an incubator at 37°C/ 5% CO_2_ in 75 or 150 cm^2^ flasks, replacing media every 2–3 days and passaging before confluence by disassociation with trypsin, washing and seeding new flasks or treatment wells. As a suspension cell line, THP-1 cells were passed directly into fresh medium.

### Cell dosing and controls

**Figure 2:**
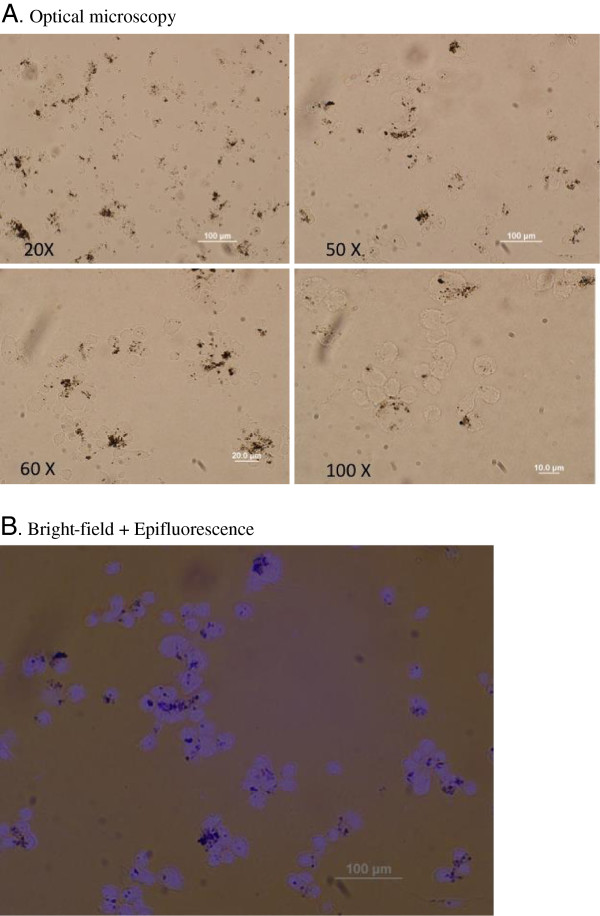
**Cellular uptake of nanoparticles by THP-1 cells at dose of 30 μg/mL nanoparticles. (A)** Optical microscopy **(B)** Bright-field + Epifluorescence.

For exposure, 5 × 10^5^ cells/mL were used in all the experiments. The primary respiratory epithelial cells were seeded into 96 well plates and allowed to recover, attach and proliferate for 24 hrs. THP-1 cells were differentiated into macrophages by culturing in the medium supplemented with 200 nM PMA for 24 hrs into 96 well plates. The media was then exchanged to fresh media without PMA. Then the cell culture media in all the cell types was replaced with 200 μl of respective fresh media containing the treatments i.e. 30, 100 and 300 μg/mL of the NP preparations (Figure 2). These concentrations were equivalent to 18, 62.5 and 180 μg/cm^2^.

The media supernatants were harvested after 6 and 24 hrs of treatment and stored at −80°C, which were later used to perform cytokine analyses using Luminex (x-Map™) technology). The cells were washed with serum free media and then trypsinized for 5 min to detach the cells from the 96 well plates. The cell suspension was centrifuged at 500×g for 10 min and washed with PBS. The cells were used to assess cell viability using typan blue (VWR, Bellefonte, PA), apoptosis via Annexin V staining, and DNA damage using the comet assay. All experiments were performed in triplicates. All the protocols were approved by Institutional Biosafety Committee of UMASS Lowell (IBC # 10-07-BEL).

### Dosimetry considerations

It is important for *in-vitro* nanotoxicology to understand how doses given to cells compare to realistic human exposure scenarios under investigation, and whenever possible, match the *in-vitro* experiments to human exposure scenarios. The deposition of aerosolized nanoparticles emitted by photocopiers in human lungs was modeled using the Multiple Path Particle Deposition (MPPD2) model [24]. The parameters used in the simulations are summarized in Table 2. Airborne nanoparticle size and mass distributions were derived from field measurements and have been reported previously [5,17]. The lung deposited surface dose [μg/m^2^] of nanoparticles was estimated by integrating the highest model derived deposition flux [μg/m^2^min] over the course of the exposure time [minutes], which in this case we estimated as that of a typical work shift, or 8 hours (480 minutes). The *in-vitro* particle suspension volume to well surface ratio [mL/m^2^] was then used to convert the surface dose of nanoparticles deposited in the lungs [μg/m^2^] to an *in-vitro* equivalent volumetric dose [μg/mL]. The underlying equation used was:

(1) $$\mathrm{dos}e_{\mathrm{in}-\mathrm{vitro},\mathrm{eq}}={\dot{m}}_{\mathrm{model}}\;x\;T_{\exp\;}x\frac{A_{\mathrm{well}}}{V_{\mathrm{admin}}}$$

**Table 2:** **Summary of parameters used in the *in vivo *lung Multiple Path Particle Deposition (MPPD2) model (MPPD2, Anjilvel and Asgharian 1995) **[24]

**Human model**	**Breathing parameters**	Airborne nanoparticle distribution*
*Functional residual capacity*: 3300.0 mL	*Tidal volume*: 625 ml	*CMD:* 34 nm
*Head volume:* 50 mL	*Breathing frequency:* 12 breaths/ min	*Geometric standard deviation*: 2.0
*Breathing route:* Nasal	*Inspiratory fraction*: 0.5	*Mass concentration*: 56.0^*^ μg/m^3^
	*Pause fraction*: 0.0	

Distribution parameters for airborne nanoparticles (size and mass) were derived from workplace monitoring data previously published by our group (Bello et al. 2012 and Khatri et al. 2012) [5,17].

* Based on the maximum peak exposures to workers operating photocopy machines of 1.2 million particles/cm^3^ (assuming effective density for aerosolized particles of 2.27 g/cm^3^). For human volunteers, average mass concentrations were ~10x lower (5.4 μg/m^3^), with average measured number concentration of 34,100 particles/cm^3^ (Khatri et al. (2012).

Where *dose*_*in-vitro,eq*_ is the *in-vitro* equivalent dose [μg/ml], *T*_*exp*_ is the total exposure time [min], *m*_*model*_ is the highest model derived mass flux to the lungs [μg/(m^2^min)], *A*_*well*_ is the surface area of the *in-vitro* well [m^2^], and *V*_*admin*_ is the volume of the media in one well [mL].

In order to convert the *in-vitro* equivalent dose, which represents the dose delivered to cells to the equivalent administered dose, the *in-vitro* sedimentation, diffusion and dosimetry (ISDD) model proposed by Hinderliter et al. [25] was utilized in order to calculate numerically the fraction of administered particles that would be deposited onto cells in a standard 96 well plate as a function of time *f*_*D*_*(t)*. Following previously described methods, particle hydrodynamic diameter (*d*_*H,*_ nm), as measured by DLS, and the measured effective density (ρ_E_, g/cm^3^) were used as inputs to the model [21]. The effective density of ENMs in physiologic fluids was measured using the volumetric centrifugation method, which was recently developed by the authors [21]. Additionally, the following parameters were used as input to the ISDD numerical model: media column height, 3.16 mm; temperature, 310 K; media density, 1.00 g/mL; and viscosity, 0.00074 Pa s [25].

### Cellular viability

Cell viability was evaluated during the treatment period (0, 6, 24 hrs) by Trypan Blue staining. One part of 0.4% trypan blue was mixed with one part cell suspension (dilution of cells). The mixture was allowed to incubate for 3 min at room temperature and then loaded on a haemocytometer to count the unstained (viable) and stained (nonviable) cells separately. Three hundred cells were counted and the procedure was replicated three times (a total of 900 cells/experiment counted). Percent viable cells were calculated accordingly as: (%) viable cells = (total number of viable cells per mL of aliquot/ total number of cells per mL of aliquot) × 100.

### Cytokine analysis

Samples were assayed using a 13-plex high sensitivity human cytokine Kit (Millipore, Billerica, MA) according to the manufacturer’s instructions. The results were analyzed using a Luminex 200IS System (Luminex Corporation, Austin, TX). Cytokine concentrations were calculated using Upstate Beadview (Temecula, CA) software. The 13 cytokines included were: Epidermal Growth Factor (EGF), Granulocyte cell stimulating factor (G-CSF), Granulocyte monocyte cell stimulating factor (GM-CSF), Interlukin-1α (IL-1α), Interlukin-1β (IL-1β), Interlukin-6 (IL-6), Interlukin-8 (IL-8), Interferon-γ (IFNγ), Monocyte Chemoattractant Protein-1 (MCP-1), Tumor Necrosis Factor α (TNF-α), Eotaxin, Fractalkine, and Vascular Endothelial Growth Factor (VEGF). Cytokines below the limit of detection (LOD) were substituted with LOD/√2 for that particular cytokine for subsequent statistical analysis.

### Interference of NP with cytokine measurements

In a separate experiment we investigated the possibility of interference of NP with cytokine measurements due to differential binding (absorption on the surface of NP) of the different cytokines to these NPs under *in-vitro* systems. For such experiments, a known concentration of recombinant human cytokine standard was added to cell culture media containing nanoparticles at concentrations of 100 μg/mL. The concentration of all 13 cytokines in this media (containing nanoparticles) and particle free cell culture media was determined using the Luminex system. These recorded cytokine concentrations were compared with the concentration of standards in assay diluents. The measured concentrations of all cytokines in the cell culture medium in the absence of NPs were close to standards in assay buffer (measured concentration = (0.95 to 1.0) × expected concentration, R^2^ = 0.998). However, the presence of nanoparticles in the medium led to a significant decrease (p < 0.5) in the measured concentration of a few cytokines (G-CSF, GM- CSF, TNF-α, IFN-γ, VEGF and IL-1α) suggesting selective adsorption of certain cytokines by nanoparticles. The three cytokines that were affected the most were VEGF (p <0.001), GM- CSF (p <0.001) and IFN-γ (p <0.01), with measured concentrations = (0.75 to 85) × expected concentration. The highest losses of 25% were found for VEGF. For other cytokines, such as EGF, IL-1β, IL-6, IL-8, Eotaxin, Fractalkine, and MCP-1, the differences were not statistically significant.

### Apoptosis

All three cell lines were treated with 30, 100 and 300 μg/mL for 6 and 24 hrs. After incubation with NPs, the cells were washed with PBS, and then stained for Annexin V using FITC Annexin V apoptosis detection kit (BD Biosciences, San Jose, CA). Additionally, cells were also co-stained with Propidium Iodide (PI, BD Biosciences, San Jose, CA). Stained cells were analyzed immediately by flow cytometry (using FACS calibus). Apoptosis was only studied in the THP-1 cell line. Flow cytometry was not possible for both types of primary cells due to their aberrant scattering properties (see Discussion for further explanation).

### DNA damage using comet assay

All three cell lines were treated with 30, 100 and 300 μg/mL for 6 and 24 hrs. The comet assay was performed for DNA damage using a commercially available kit (Cell Biolabs, San Diego, CA). All the steps were followed according to the protocol described in the kit with the only modification being regular microscope slides were used instead of slides provided with the kit. The slides were coated with 1% agarose and dried overnight at room temperature before use. After performing the assay, the slides were stained with vista green (Cell Biolabs, San Diego, CA) and viewed using epifluorescence microscopy with an FITC filter. The cell images were analyzed using CASP software (Downloaded from www. casplab.com). Among the comet parameters we report the percent of DNA in the tail (tail DNA%) as a marker of DNA damage [26]. Two hundred comets were scored randomly for each concentration and time point. All experiments were performed in triplicate.

### Transcriptional analysis of key biomarkers indicative of inflammation, apoptosis, DNA damage and oxidative damage

Gene expression level changes of key genes involved in cellular inflammation, apoptosis, DNA damage and oxidative damage were analyzed in THP-1 cells at a sub-cytotoxic dose of 5 μg/mL (>95% of cells were alive based on independent minimum inhibitory concentration tests). 5 × 10^5^/mL of THP-1 cells were seeded in 6-well plates (Costar, 4 mL per well) for 24 h in RPMI1640 medium with 10% FBS and then differentiated into macrophages by PMA for another 24 h induction as previously described. The media was then exchanged to fresh RPMI1640 medium with 1% BSA, without PMA or FBS. NPs prepared in RPMI1640 medium with 1% BSA were added into cells with final concentration of 5 μg/mL for 6 hrs exposure. RPMI1640 medium with 1% BSA only was used as untreated control. RNA was then extracted form NP-exposed cells using one step RNA reagent (BS410A, Bio Basic Inc., Canada) and reverse transcribed to cDNA by Verso cDNA Synthesis Kit (Thermo Scientific, US). Q-PCR was performed using SYBR Green Supermix (Bio-Rad, US) on iQ™5 Multicolor Real-Time PCR detection system (Bio-Rad, US). PCR primers targeting the 10 selected genes indicating inflammation, apoptosis, DNA damage and oxidative damage (Table 3) were obtained based on literature review and the NCBI database, with housekeeping genes GAPDH and 18 s RNA used as internal control. The primers are listed in (Table 3). The changes in gene expression was normalized by the geometric average of GAPDH and 18 s RNA based on multiple internal control normalization [27]. Fold change due to treatment compared to untreated control was determined as 2^-ΔΔCT^. The experiment was performed in triplicate.

**Table 3:** Genes and primers for gene expression analysis in THP-1 cells used in this study

**Phenotypic endpoints (Stress type)**	**Gene**	**Sequence**	**Product size (bp)**	**Tm (°C)**	**Resource**
Inflame-mation	TNF-α	F:CGAGTCTGGGCAGGTCTACTTT	299	60	UniSTS:271115
		R:AAGCTGTAGGCCCCAGTGAGTT			
	IL-1β	F:AGTCAGCTCTCTCCTTTCAGG	229	56	G10509
		R:CTTGCCCCCTTTGAATAAAT			
Apoptosis	P53	F: TGTGGGATGGGGTGAGATTTC	147	60	UniSTS:151711
		R: CTGTTGGTCGGTGGGTTG			
	CASP3	F:TATGGTTTTGTGATGTTTGTCC	195	56	G10724
		R:TAGATCCAGGGGCATTGTAG			
	CASP8	F:ATGGACTTCAGCAGAAATCTT	250	58	X98172
		R:TCTAGTGTTTAGGTAGGTAATCAGC			
DNA damage (double strand break)	KU70	F: TTTTTTTGGTTGATGCCTCC	135	60	D51189 G28341
		R: CCAAGAGATCTCGATCACTGC			
	RAD51	F: TTAAAAACCTTAAGTGCTGCAGC	121	56	UniSTS: 57459
		R: GGATTATCTTGAGTTAGTCTTAGC			
Oxidative damage	HO1	F: CATGACACCAAGGACCAGA	155	60	[28]
		R: AGTGTAAGGACCCATCGGAG			
	SOD1	F:CCATCTGTGATTTAAGTCTGGC	253	60	N32033
		R:AAACATTCCCTTGGATGTAGTCTG			
	GPX1	F: GCCTGGGCTCCCTGCGGGGCAAGG	470	60	[29]
		R: TACGAAAGCGGCGGCTGTACCTGCG			
Internal control	GAPDH	F: CCATGTTCGTCATGGGTGTGAACCA	251	60	[30]
		R: GCCAGTAGAGGCAGGGATGATGTTC			
	18S rRNA	F: GTAACCCGTTGAACCCCATT	151	60	[31]
		R: CAGGGACTTAATCAACGCAA			

F: forward primer; R: reverse primer. The original primers for the selected genes were acquired through GenBank Accession number/UniSTS number or from literature.

### Statistical analysis

Statistical differences between groups were tested via repeated measures two-way ANOVA, Turkey multiple comparison test, in Graphpad prism software 5 Inc**,** CA, USA. All experiments were repeated three independent times (n = 3). Results were considered statistically significant at p < 0.05 (notation, p < 0.001, ***, p < 0.01, **, and p < 0.05, *). Results are expressed as mean ± SE or otherwise stated. Values less than LOD were substituted with LOD/√2.

## Results

### Dosimetry considerations

**Figure 3:**
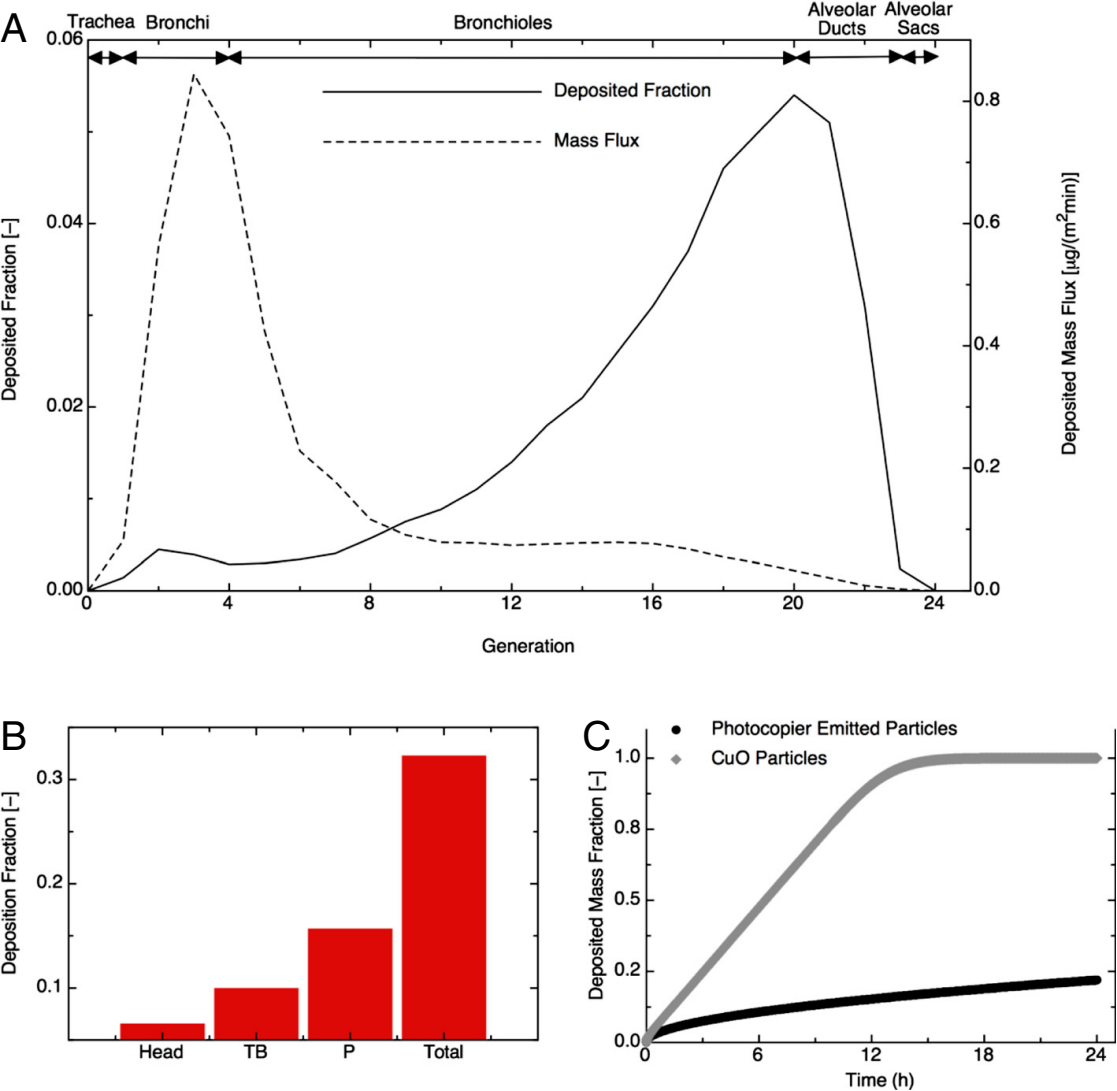
**Modeled deposited mass fraction and the mass flux (μg/m**^**2**^**) for the different regions of the lung (shown as generation number). (A)** Model calculations of deposition mass flux (left axis) and deposition fraction (right axis) as a function of generation number. **(B)** Deposition fraction in total lung for head, tracheobronchial region (TB) and pulmonary region (P). **(C) ***In-vitro dosimetry*: delivered mass fraction as a function of time for photocopier emitted - and CuO nanoparticles.

Figure 3 shows the modeled deposited mass fraction and the mass flux (μg/m^2^) for the different regions of the lung (shown as generation number). The model predicts deposition of most particle mass deeper in the pulmonary region of the human lungs (~15% mass, Figure 3B). This is somewhat expected given the small size (34 nm median count diameter) of the photocopier emitted particles. By comparison, ~10% mass fraction is deposited in the tracheobronchial region and ~6% in the head-airway region. Furthermore, based on a deposition mass flux of 0.844 μg/m^2^min (Figure 3A) and an exposure time of 480 min (equivalent to an 8 hour work shift), a lung surface dose of 405 μg/m^2^ was estimated.

Equation 1 was used to calculate the equivalent *in-vitro* dose delivered to cells, which was found to be 0.13 μg/mL (well diameter = 6.35 mm, and media volume in a well = 0.1 mL). However, the *in-vitro* dose delivered to cell is not the same as the administered dose [21,25] as only a fraction of the administered mass reaches the cells at the bottom of the well over time. The recently developed ISDD numerical model was used here to estimate the fraction of administered dose actually delivered to cells over time (Figure 3C) [21,25]. As shown in Figure 3C, following 6 hours of exposure, only ~10% of the photocopier emitted nanoparticles and 47.5% of the CuO (34 nm primary size) reference particles would be delivered to cells. At 24 hours of exposure only ~20% of the photocopier emitted nanoparticles, and 100% of the CuO particles were delivered to cells. Therefore, ~5 (at 24 hrs) and 10 times (at 6 hrs) higher administered doses are required for photocopier emitted nanoparticles to achieve a target delivered dose derived from model deposition calculations. An administered dose of 0.58 μg/mL (i.e. ~5 × higher) for the photocopier emitted particles is required to achieve the equivalent *in-vitro* delivered to cell dose of 0.13 μg/mL following 24 hours of exposure, which matches the estimated equivalent dose of 405 μg/m^2^ for particles deposited in the human lung. At 6 hr, the equivalent administered dose is estimated at 1.3 μg/mL (i.e. 10× higher).

The effective dose of photocopier-emitted nanoparticles delivered to cells would be 0.1× (at 6 hrs) and 0.2× (at 24 hrs) of the administered dose (30, 100, 300 μg/mL), respectively. At 6 hrs, these doses would be: 3, 10, and 30 μg/mL. For the comparative CuO nanoparticles, the effective doses at 6 hrs would be ~0.5 × administered dose, or 15, 50 and 150 μg/mL). At 24 hrs, the effective doses of photocopier emitted nanoparticles delivered to cells would be 6, 20, and 60 μg/mL, whereas for CuO nanoparticles, 30, 100, 300 μg/mL.

### Cellular viability

**Figure 4:**
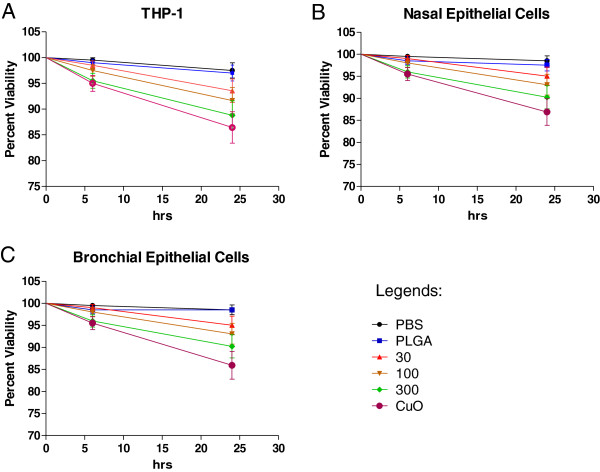
**Percent viability of cells determined using typan blue staining at different time points and treatment concentrations.** Cell viability results depicted as a function of administered dose in three cell types **(A)** THP-1 cell line, **(B)** nasal epithelial cells and **(C)** small airway epithelial cells. All values are represented as mean ± SE.

Cell viability results as a function of the administered dose are presented in Figure 4. A modest but significant dose and time dependent effect on cell viability was observed at both treatment time points in all of the three cell types and the viability curves were significantly different from both PBS and PLGA negative control at 100 (p < 0.05) and 300 μg/mL doses (p < 0.01). In the THP-1 cell line, the cell viability at 6 hrs was 98.50 ± 0.85%, 97.50 ± 1.10% and 95.50 ± 1.46% at 30, 100 and 300 μg/mL doses, respectively, and at 24 hrs of treatment time point the cell viability was 93.6 ± 2.4, 91.7 ± 2.5 and 88.8 ± 2.8, respectively (Figure 4A). In primary nasal epithelial cells, the cell viability at three treatment concentrations was 99.0 ± 0.7%, 98.00 ± 0.98%, and 96.00 ± 1.38% at 6 hr time point and 95.0 ± 2.0%, 93.1 ± 2.3% and 90.2 ± 2.6% at 24 hrs of treatment time point (Figure 4B). In small airway epithelial cells, the cell viability at 6 hrs was 99.0 ± 0.7%, 98.0 ± 1.0% and 96.0 ± 1.4%, and at 24 hrs of treatment time point the cell viability was 95.04 ± 2.05%, 93.10 ± 2.33% and 90.24 ± 2.62% at three treatment concentrations, respectively (Figure 4C). Compared to photocopier-emitted nanoparticles, CuO nanoparticles exhibited lower cell viability on an equal administered mass dose, especially at 24 hrs (85-88% for all three cell types).

### Cytokine release in three cell types after NP treatment

#### THP-1 cell line

**Figure 5:**
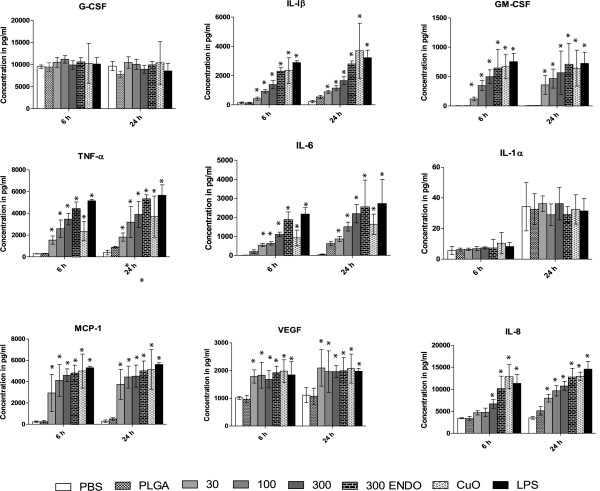
**Levels of various cytokines at 6 and 24 h after nanoparticle treatment in THP-1 cell line.** The concentrations of various cytokines measured in cell culture supernatant after nanoparticle treatment at different time points. All values are in picograms and are represented as mean± SE.

The concentration of several cytokines, namely GM-CSF, IL-1β, IL-6, IL-8, MCP-1 and TNF-α, increased in a dose and time dependent manner, and all of them were significantly elevated at the administered 300 μg/mL NP concentration relative to both PBS and PLGA negative controls at both 6 h and 24 h time points (p < 0.001) (Figure 5). At 100 μg/mL, cytokines, IL-8, MCP-1 and TNF-α were significantly higher at 24 hr compared to control (p < 0.5). However, only GM-CSF, IL-6, MCP-1 and TNF-α were elevated at 6 hr (p < 0.5) (Table 4). In the case of VEGF, although no dose dependent effect was observed, its concentration was significantly elevated at all three administered doses at both 6 h and 24 h time points (p < 0.01).

**Table 4:** Comparative summary of all cytokines that were unregulated in three cell types and nasal lavage of health volunteers post-exposure (Khatri et al. 2012) in response to nanoparticles emitted from photocopiers

**Test system**	***In Vitro***						***In Vivo***
	**THP-1**		**Nasal epithelial cells**		**Small airway epithelial cells**		**Nasal lavage of healthy volunteers (Khatri et al. 2012)**
**Cytokine**	**↑**	**Dose information**	**↑**	**Dose information**	**↑**	**Dose information**	
		**(μg/mL)**		**(μg/mL)**		**(μg/mL)**	
**IL-8**	***	6 hr, only at 300	***	6 h, all doses	***	6 h, all doses	***
		24 hr, all doses		24 h, all doses		24 h, all doses	
**IL-6**	***	6 hr, only at 300	-	-	-	-	***
		24 hr, 100 & 300					
**IL-1β**	**	6 hr, 300	-	-	*	24 hr, only at 300	***
		24 hr, 300					
**GM-CSF**	**	6 hr, 300	-	-	*	24 hr, only at 100	-
		24 hr, 100 & 300					
**TNF-α**	***	6 hr, 100 & 300	*	24 hr, only at 300	-	-	***
		24 hr, 100 & 300					
**MCP-1**	***	6 h, all doses	-	-	-	-	***
		24 h, all doses					
**VEGF**	*	6 hr, 100	**	24 hr, 30 & 100	**	24 hr, 30 & 100	***
		24 hr, all doses					
**IL-1α**	-	-	***	24 hr, 100 & 300	*	24 hr, 100 & 300	-
**G-CSF**	-	-	-	-	-	-	***
**Fractalkine**	**	24 hr, 100 & 300	-	-	-	-	***
**EGF**	-	-	***	6 hr, 300	***	24 h, all doses	***
				24 hr, all doses			
**IFN-γ**	-	-	*	24 hr, 30 & 100	-	-	-

**↑**, Upregulated, p < 0.001 (***), p < 0.01 (**), p < 0.05 (*), ‘-‘, statistically non-significant. The two epithelial cell types are primary human cells. Comparisons are relative to negative controls.

Cells exposed to nanoparticles without Polymyxin B treatment (administered dose of 300 μg/mL) produced slightly higher levels of cytokines (GM-CSF, IL-6, IL-8, MCP-1, TNF-α and VEGF) than the corresponding Polymyxin B treated nanoparticles at both time points. These differences, however, were not statistically significant.

PBS and PLGA beads were used as negative controls. The PLGA beads did not show any significant difference in cytokine concentration as compared to PBS for all cytokines. By comparison, the positive control CuO nanoparticles caused a significant increase (p < 0.01) in GM-CSF, IL-1β, IL-6, IL-8, MCP-1, VEGF and TNF-α concentrations at both 6 h and 24 h as compared to both negative controls, and these cytokine concentrations were higher than those induced by photocopier-emitted NPs, in case of most of the cytokines. The cells treated with 1 μg/mL LPS (also a positive control) showed a significant increase relative to both negative control (p < 0.001) in GM-CSF, IL-1β, IL-6, IL-8, MCP-1, VEGF, Fractalkine (not shown in Figure 5) and TNF-α. There was no significant difference between the levels of IL-1α and G-CSF in the negative control and NP treated groups at all concentrations at both time points. Additionally, Eotaxin, EGF and IFN-γ were not included in the final analysis because the majority of samples were below the method’s limit of detection (LOD), which were were 1.2 pg/ mL for Eotaxin, 2.7 pg/mL for EGF, and 0.1 pg/mL for IFN-γ.

#### Primary nasal and small airway epithelial cells

**Figure 6:**
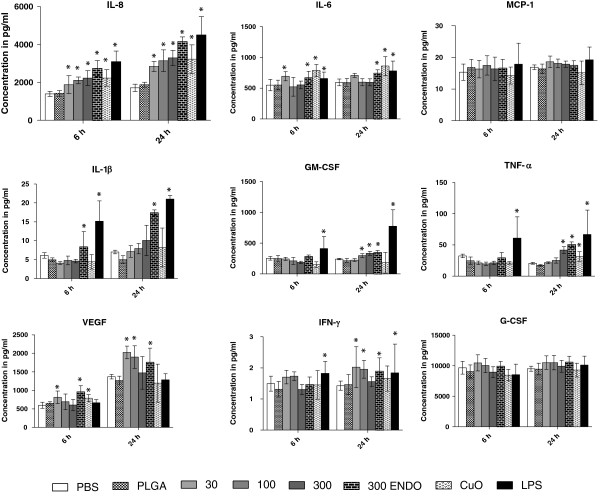
**Levels of various cytokines at 6 and 24 h after nanoparticle treatment in primary nasal epithelial cells.** The concentrations of various cytokines measured in cell culture supernatant after nanoparticle treatment at different time points. All values are in picograms and represented as mean± SE.

The concentrations of cytokines produced by both types of primary cells were, as expected, much lower than those in the THP-1 cell lines (summarized in Table 4). They also were generally higher at 24 hrs than at 6 hrs (Figure 6). In primary nasal epithelial cells, there was a statistically significant increase (p < 0.001) in IL-8 relative to the both PBS and PLGA negative control at all three NP administered concentrations (30, 100 and 300 μg/mL) at 24 h and for 300 μg/mL at 6 hrs. Other cytokines such as IL-6, VEGF, TNF-α, IFNγ, IL-1α and EGF (not shown) were significantly elevated at only certain treatment concentrations and time points (Table 4, Figure 6). There was no significant increase in the levels of MCP-1, IL-1β, G-CSF, Fractalkine (not shown) and GM-CSF for any dose or treatment groups at either time points. Eotaxin was dropped from the final analysis as its concentration was below the Luminex limit of detection (1.2 pg/mL) in all of the samples. The highest administered NP dose of 300 μg/mL without Polymyxin B treatment induced significantly higher levels (p < 0.05) relative to the same dose treated with Polymixn B for the cytokines IL-6, IL-8, IL-1α, TNF-α, IFN-γ and IL-1β. The magnitude of this effect was cytokine dependent and overall modest, except for IL-1β, for which the effect was more prominent. However, the overall levels of IL-1β were also very low (<20 pg/mL).

**Figure 7:**
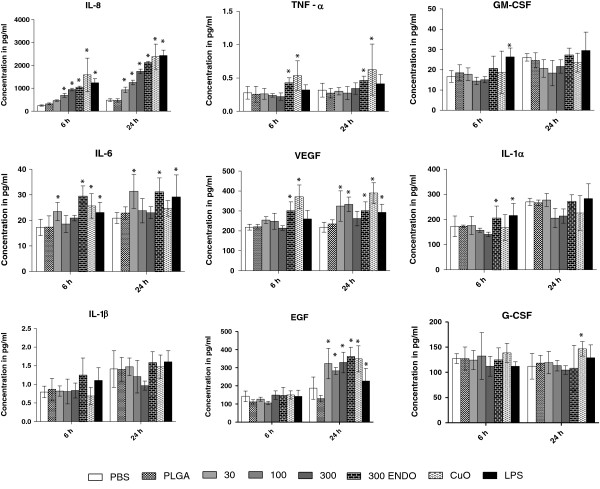
**Levels of various cytokines at 6 and 24 h after nanoparticle treatment in primary small airway epithelial cells.** The concentrations of various cytokines measured in cell culture supernatant after nanoparticle treatment at different time points. All values are in picograms and represented as mean± SE.

In the primary small airway epithelial cells, IL-8 was significantly increased (p < 0.05) relative to the negative controls at all NP doses at 24 h and at 6 hr, with the exception of the lowest NP concentration (30 μg/mL) (Figure 7). Most of the cytokines such as IL-6, IL-1β, EGF, IL-1α and VEGF were significantly elevated at only certain treatment concentrations and time points (Table 4). Other cytokines, including G-CSF, GM-CSF and TNF-α, did not show any significant change at any NP dose or time point. Four cytokines, namely Eotaxin, Fractalkine, MCP-1 and IFN-γ, were not included in the final analysis as most of the values were below the system’s limit of detection (LOD’s for Fractalkine, MCP-1 and IFN-γ were, 6.0, 0.9 and 0.1 pg/mL, respectively).

As with the nasal cells, the effect of endotoxins on inflammation of primary small airway epithelial cells was studied by comparing the same NP dose (300 μg/mL) with and without Polymyxin B treatment. Select cytokines, namely TNF-α, IL-6, VEGF and IL-1β, were slightly higher (p < 0.05) in the group without Polymyxin B treatment. No significant difference was observed between the negative controls PBS and PLGA for any of the cytokines for either the 6 hr or 24 hr measurements in any of the primary cell types. Significantly higher cytokine levels were observed for the positive control copper oxide nanoparticles and LPS as compared to negative controls (p < 0.05) for all cytokines in both cell types. Similar to results obtained from THP-1 cells, these cytokine concentrations were higher than the corresponding treatments of NPs from the photocopier. Note that due to interferences with nanoparticles at high doses, cytokine concentrations are likely underestimated.

#### DNA damage by the comet assay

**Figure 8:**
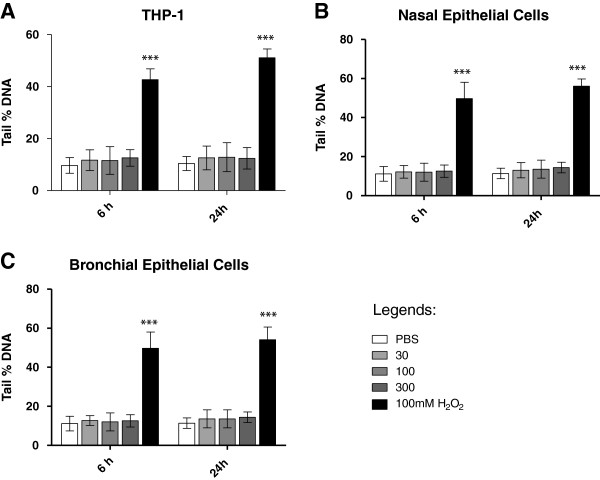
**Percentage DNA damage studied using comet assay in all three cell types.** Percent of DNA in the tail (tail DNA%) was used as a marker of DNA damage in all three cell types **(A)** THP-1 cell line, **(B)** nasal epithelial cells and **(C)** small airway epithelial cells. All values are depicted as mean± SE.

Comet assay results are summarized in Figure 8. No significant genotoxicity was observed at any dose and time point for all the three cell types. Significant DNA (p < 0.001) damage was observed in positive control cells treated with 100 mM H_2_O_2_ (Figure 6).

#### Cell apoptosis

**Figure 9:**
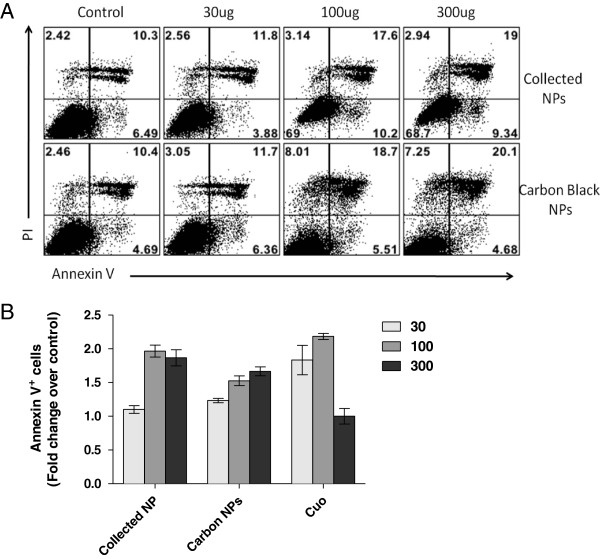
**Cell apoptosis using AnnexinV and PI at different NP treatment concentration. (A)** Cell apoptosis in THP-cell lines after 24 h of NP treatment, **(B)** Fold change in Annexin V + cells over control. Cells were analyzed using flow cytometry. All values are represented as mean± SE.

Despite the lack of forming tail DNA in the Comet assay, a significant number of THP-1 cells exposed to different concentrations of NPs for 24 hrs exhibited apoptosis as measured by Annexin V staining (Figure 8A and B). Compared to unexposed THP-1 cells (negative control), up to two-fold increase in the frequencies of Annexin V positive cells was recorded when cells were exposed to 100 μg/mL (Figure 8B). At the highest administered NP dose of 300 μg/mL, no further significant increase in the frequency of cells stained with Annexin V was observed. Similar results were obtained when cells were exposed to various concentrations of carbon black NPs (N550, 44 nm primary size). Cells exposed to CuO nanoparticles, at comparable concentrations, exhibited greater levels of apoptosis (Figure 8B). However, the reduced frequencies of Annexin V^+^ cells were recorded when cells were exposed to 300 μg/mL of CuO. This anomaly at 300 μg/mL NP may be caused by NP interfere with fluorescence detection systems, as well as there may a possible direct interactions of NPs with the AnnexinV or PI as noted by other authors [32-34]. We also attempted to analyze Annexin V levels in primary and small airway epithelial cells, however we consistently obtained aberrant side scattering results in the flow cytometer and therefore were unable to analyze apoptosis caused by NPs in these cells. This may be due to the fact that these epithelial cells are not very efficient in internalizing NPs as compared to THP-1 cells, leaving the majority of NPs attached to the cell membrane (confirmed by imaging) that could not be removed even after multiple washing steps before staining, leading to interference with the forward and side scatter light of the detection system. Despite this issue, the combined results in Figure 9 suggest that collected NP from photocopiers induced apoptosis of monocytic THP-1 cells.

#### Transcriptional analysis

**Figure 10:**
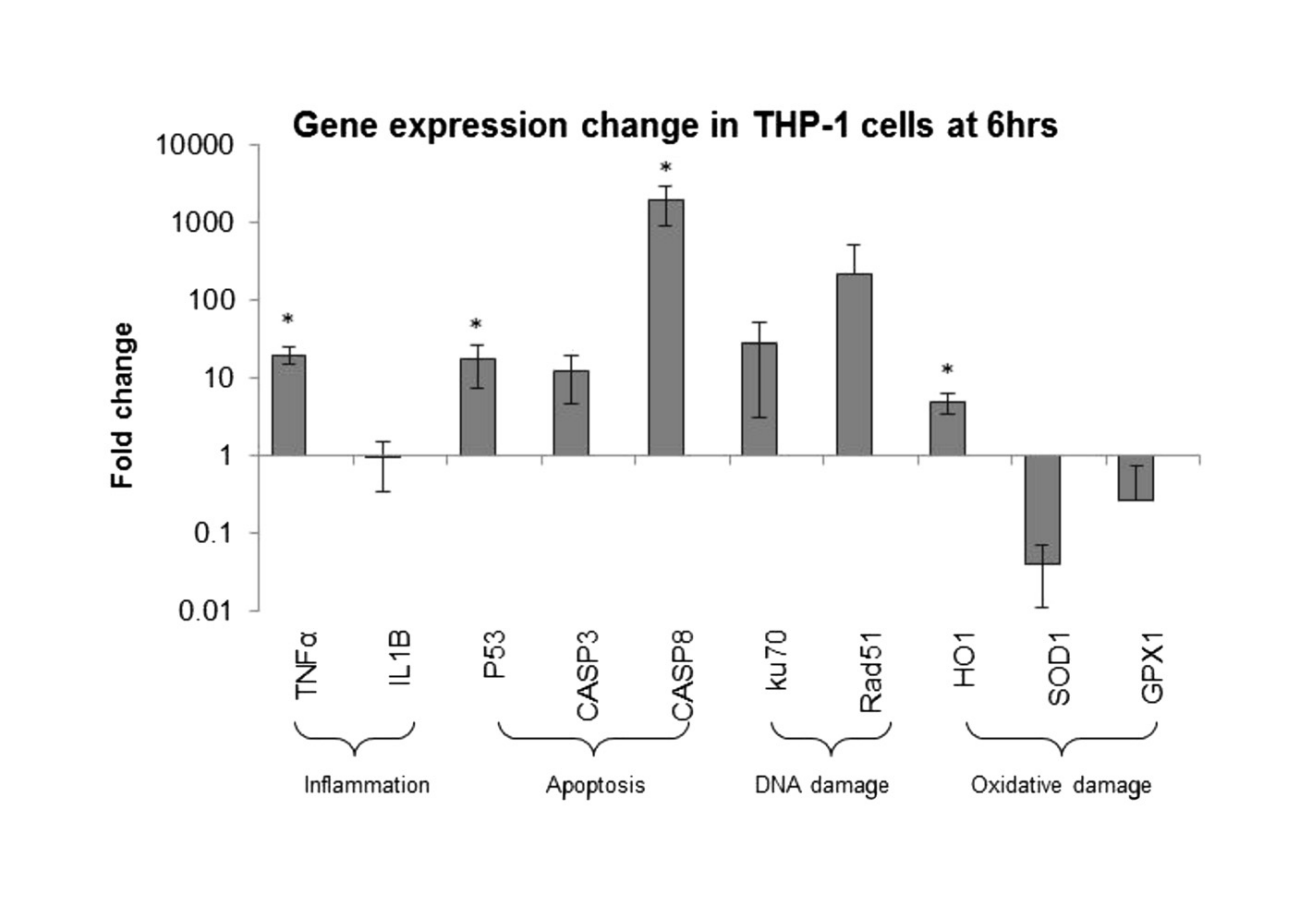
**Gene expression change in THP-1 cells after 6 hr exposure of 5 μg/mL nanoparticles collected from photocopiers.** RT-qPCR indicated gene alteration by fold change (Y-axis), *compared to untreated control, *p < 0.05. n = 3.

A lower concentration of NP (5 μg/mL) was used for gene expression test in THP-1 cells at sub-cytotoxic dose (>95% cells survived). Strong inflammation and apoptosis effect related to oxidative damage was observed for 6 hr exposure (Figure 10), which was consistent with phenotypic endpoints observed in this study. For inflammation, pro-inflammatory cytokine TNF-α expression was significantly up-regulated for THP-1 cells exposed to NPs compared to negative control consistent with cytokine release. However, no significant difference was observed for IL-1β expression, which may be due to the lower concentration for transcriptional analysis, as the significant elevation of IL-1β release was only observed at 300 μg/mL. For DNA damage, both Ku70 and Rad51 for DNA double strand break repair were up regulated compared to untreated control, although not statistically significant (p = 0.23), indicating potential DNA damage may be induced. However, no significant genotoxicity was observed at any dose and time point in the comet assay. For apoptosis, key genes P53 (controlling cell cycle and apoptosis) [35] and CASP8 (mediating mitochondrial damage in the Fas pathway of apoptosis) [36] were significantly up regulated, suggesting apoptosis was induced and they were consistent with the Annexin V staining test. Significant up-regulation of HO1, a stress-responsive protein induced by various oxidative agents [37], suggested oxidative damage has induced, which is consistent with the previous report [17]. The down-regulation of copper-zinc superoxide dismutase (SOD1) and glutathione peroxidase (GPX1) also suggested that ROS metabolism might be modulated to increase ROS generation [38].

## Discussion

This study is the first investigation of cytotoxicity of real-world nanoparticles emitted from photocopiers and the fifth in a series of articles on the properties and toxicology of such nanoparticles. In our first study, we described the physicochemical and morphological properties of these nanoparticles and established their complex chemistry as a mixture of metal oxides, and poorly-understood organic fraction [5]. In the second, we showed that the nanoparticles caused a 2–10 fold increase in inflammatory cytokines, inflammatory cell influx, and total protein in nasal lavages, and oxidative stress marker 8-Oxo-2′-deoxyguanosine *(8-OH-dG)* in the urine of human volunteers after acute exposure to such nanoparticles in a photocopy center at modest exposure levels (average 34,000 particles/cm^3^) [17]. Furthermore, these effects cleared slowly over the next 24–36 hrs post-exposure [17].

In another publication by the authors [39], size selective PM_0.1_ and PM_0.1–2.5_ size fractions from the same copy center were instilled intratracheally in Blab/c mice [39] in order to assess lung injury and inflammation of inhaled PM. Mice instilled with PM_0.1_ size fraction had significant increases in neutrophil number, lactate dehydrogenase and albumin compared to vehicle control. Likewise, several pro-inflammatory cytokines in the bronchoalveolar lavage fluid, including IL-1α, VEGF, and G-CSF, were also elevated in mice exposed to PM 0.1 compared to other groups. Comparatively, the nanoscale fraction was considerably more potent than the PM_0.1–2.5_ fraction, and comparable to the reference/comparator welding fumes, in inducing these effects [39].

Furthermore, in a companion paper of a similar design to the current *in-vitro* work, we found that several cytokines such as IL-6, IL-8, TNF-α and IL-1β were significantly elevated in THP-1 cells following dosing with the PM_0.25–2.0_ μm fraction [40]. Only IL-8 was significantly elevated in the primary nasal and small airway cells. THP-1 cells also underwent apoptosis in a dose dependent manner. However, no significant differences were found in the extent of DNA damage at any time point [40]. Similar to Pirella’s study in mice [39], the PM_0.25–2.0_ μm fraction in Khatri et al. [40] was significantly less potent in inducing a response *in-vitro* (e.g. inflammation and apoptosis) than the nanoscale fraction tested in this study.

Here we aimed at investigating cytotoxic properties of the nanoscale fraction of copier-emitted nanoparticles in three cell lines, relevant to respiratory exposures in humans: THP-1 cell as surrogated for human lung macrophages; primary human nasal epithelial cells, for upper airways; and primary human small airways epithelial cells for deeper airways. We purposefully focused on the production of inflammatory cytokines / chemokines by these cells due to their important mediatory roles in inflammation, insights into possible molecular mechanisms, and to match them with cytokines in nasal lavage of human volunteers. Inflammation, genotoxicity, and oxidative stress are frequently reported endpoints in several human [10-14,17,41] and in the limited *in-vitro* studies on printer-emitted nanoparticles [15,16]. In addition to these end points, we also investigated apoptosis, DNA damage and supplemented further by gene expression data for key markers of inflammation, oxidative stress, genotoxicity, and apoptosis at sub-cytotoxic concentrations in THP-1 cells. DNA damage and aberrant immune responses have been frequently reported in photocopier operators, together with evidence of cellular apoptosis [11,14]. Consistent with these previous results, we found that NPs emitted from photocopiers are capable of inducing the release of several pro-inflammatory cytokines, *in-vitro* and, at least in the case of monocytic cells, induce apoptosis in a time and dose-dependent fashion, but with modest acute cytotoxicity to the cells. In THP-1 cells, gene expression data at 5 μ/mL administered dose and 6 hr exposure duration, confirmed up-regulation of key genes controlling inflammation (TNF-α), apoptosis (p53, CASP8), and oxidative stress (HO1), further supporting the likely involvement of oxidative stress in these responses. These results provide important insights into the toxicological potential and the type of immune responses elicited by these nanoparticles.

Cytokine/Chemokine expression was (as one would expect) cell type dependent, with the monocytic THP-1 cell line being more sensitive to NP exposure than the two primary respiratory epithelial cells. The measured levels of secreted cytokines by the THP-1 cell type were about an order of magnitude higher than in the primary epithelial cells. The higher sensitivity of THP-1 cells may be partly attributed to the fact that we used PMA-differentiated THP-1 macrophages, which have greater ability to engulf particles through phagocytic mechanisms, thereby increasing cellular responses to NPs [42,43]. The primary nasal and small airway epithelial cells in general secreted low cytokine levels (5 pg/mL-1 ng/mL range). Even for IL-8, which had the highest concentrations of 5 ng/mL were ~3 times lower than in THP-1 cells. IL-8 is a chemokine secreted by epithelial cells and is known to induce chemotaxis of inflammatory cells to sites of action and thus has a prominent role in the development of inflammation. This may due to the lack of simultaneous multiple cell type interactions in response to nanoparticles, typical of the living tissues in whole organisms [33]. In addition, the reduced phagocytotic capacity of primary epithelial cells compared to macrophage-like cells, may result in smaller internalized doses in the epithelial cells, causing less activation of the downstream signaling pathways leading to cytokine release and less cytokine production [44,45]. At the highest administered NP doses, interaction of selected cytokines (such as GM-CSF, VEGF and TNF-α) with NPs might have lead to significant cytokine losses and, as a consequence, underestimation of their true concentration, due to surface absorption by NP and possibly also due to induced conformational changes. Other researchers have also reported cytokine losses due to surface absorption by NPs in *in-vitro* conditions [29,35].

The types of secreted cytokines *in-vitro* were similar to those found *in vivo* in the nasal lavage (NL) fluid of healthy subjects after acute (6 hrs) NP exposure in the photocopy center environment [17]. Several cytokines, namely IL-6, IL-8 TNFα, IL-1β, G-CSF, EGF, IL-10, MCP-1, Fractalkine and VEGF, were elevated *in vivo* in the NL fluid of the participating volunteers. Several of these cytokines, specifically GM-CSF, IL-1β, IL-6, IL-8, MCP-1, TNF-α and VEGF, were also expressed *in-vitro* in the THP-1 cell line in the current study. Fewer of these, namely IL-8, TNF-α, EGF and IL-1α, were induced in both primary epithelial cell lines (summarized in Table 4). Of note, IFN-γ was overexpressed only in primary nasal epithelial cells. Another cytokine, namely G-CSF is also worth noting because it is overexpressed in human NL but not *in-vitro*. That might be due the reason that a complex activation machinery requiring multiple interactions in live tissue is required for the expression of these cytokines, which is absent in a single cell type [46]. Overexpression of IL-1α in both primary cell lines *in-vitro* but not in NL may be a reflection of higher *in-vitro* doses. Taken together, the *in-vitro* cytokine/chemokine findings are consistent with our previous human volunteers study [17] and reinforce our earlier conclusion that copier-emitted nanoparticles are directly responsible for the induction of pro-inflammatory responses.

Most of the cytokines secreted in the present study play a major role in the development of inflammation, which is a gateway to many chronic diseases. Pro-inflammatory cytokines such as TNF-α, IL-1β and GM-CSF play important roles in amplification and maintenance of airway inflammation and are known to be key players in the clinical exacerbations and chronicity of bronchial asthma, an inflammatory disorder of the airways [47]. Additionally, the production of chemokines such as IL-8 from alveolar macrophages and epithelial cells is thought to support neutrophil migration into the airspaces of the lung in idiopathic pulmonary fibrosis (IPF) and subsequent alveolar damage and tissue fibrosis [48-50]. VEGF plays important roles in angiogenesis (growth of new blood vessels) and increased vascular permeability [51]. The chemokine MCP-1 is involved in the recruitment of monocytes to sites of injury and infection and elevated levels play a key role in the clinical course of interstitial lung disease [52,53]. Therefore, the results from the present study confirm the ability of NPs from photocopiers to trigger inflammatory responses in humans, which upon chronic exposures may, in turn, lead to or intensify any preexisting respiratory condition such as asthma or chronic obstructive pulmonary disease (COPD).

It is important to acknowledge that direct quantitative comparisons between *in vivo* and *in-vitro* are difficult to make, because of dosimetry consideration and the simplicity of the single cell *in-vitro* models. As our human deposition flux models employed here indicate, the estimated *in vivo* dose to the lungs using the highest measured exposures to workers would correspond to 0.13 μg/mL in the *in-vitro* system. After correcting for the dose delivered to cells, the *in-vitro* delivered dose (3–30 μg/mL at 6 hrs and 6–60 μg/mL at 24 hrs) is over an order of magnitude higher than *in vivo* dose to the deep airway. The nasal cavity may receive much higher doses than the deep airway because its total surface area is disproportionately smaller (~150 cm^2^ for the nasal cavity vs. 120 m^2^ for the lungs) than the total NP deposition fractions (6% in nasal cavity vs. ~33% in the deep airways, Figure 3) and the *in-vitro* delivered NP doses are more comparable to *in vivo* upper airway doses. However, *in vivo,* the effective dose of nanoparticles reaching the epithelial cells is smaller than the deposited dose, partly due to the active clearance by macrophages and mucociliary transport, and party due to the barrier provided by the mucus layer, the lung surfactants and its antioxidants.

Copier-emitted nanoparticles also induced cell apoptosis in THP-1 cells, which was shown to occur in a dose- and time-dependent manner. The Annexin V staining results were confirmed by gene expression analysis at a much lower dose (5 μg/mL at 6 hrs), showing up-regulation of CASP8 and p53 but not CASP3, implicating the Fas pathway. At high NP concentrations, especially at 300 ng/mL, interference with Annexin V staining is apparent for both copier-emitted and CuO NPs. This is consistent with previous reports in which NPs have been shown to interact with PI [54], thereby over-representing the results in detection of necrotic cells, which might be the case with the present study, as we observe higher AnnexinV/PI positive cells at all concentration as compared to cell death results obtained using trypan blue staining. Furthermore, we were unsuccessful at studying apoptosis in primary nasal and small airway epithelial cells after NP treatment due to aberrant scattering properties of these cells in the flow cytometer. Poor internalization of NPs by the primary epithelial cells compared to THP-1, would favor attachment of large amounts of NPs to the outside cell membrane, which could not be removed with repeated cell washing, leading to interferences with the forward and side scatter light of the detection system [33,55].

There are only few relevant studies on nanoparticles from photocopy equipment to compare our results with. Genotoxicity has been a particular focus on the debate of (nanoparticle and toner) emissions from printing and photocopy equipment and the subject of several past studies [16,56,57]. Gminski et al. [16] used DMSO extracts from toner powders to dope A549 lung cells and observed genotoxic effects [16]. The chemical composition of the DMSO extracts was not characterized. More recently, Tang et al. [15] used an air-to-liquid system to dope cells with actual nanoparticles emitted from different printers. They reported that the printer emissions were found to cause genotoxicity, however no cytotoxicity was observed. These reports did not provide information about inflammatory properties caused by the printer emissions or the toner. These studies, point to the need to consider variability in the chemical composition of emissions across different toner formulations/manufacturers. In the present study, we did not observe any significant DNA damage based on comet assay in any of the three cell types for test nanoparticles originating from one manufacturer at all concentrations tested. However, high level up-regulation for DNA double strand break genes was observed in THP-1 cells at 6 h with 5 μg/mL, indicating DNA damage potential consistent with previous report [11,12,17]. One possible explanations for this discrepancy is that the extracts used in the past studies may differ in chemical composition from airborne fractions in significant ways, i.e. the organic fraction may be over-enriched with certain organic components, and lacks the inorganic component. Large variations in chemical composition of emitted nanoparticles between different manufacturers may exist, further contributing to discrepancies between different studies. As mentioned in the introduction, few human studies also reported genotoxic effects in blood cells and significant chromosomal aberrations in lymphocytes of copy center workers [11,12]. Previously, we found increased levels of urinary *8-OH-dG*, a marker of systemic oxidative DNA damage [17], in human volunteers following a single exposure. Gene expression in this study also confirmed oxidative damage in THP-1 cells, reflected in the up-regulation of HO1, and down-regulation of SOD1 and GPX1. The discrepancy between high oxidative stress in humans and lack of *in-vitro* genotoxicity may be due to aging of NP aerosols in the ambient environment or, perhaps *8-OH-dG* reflected increased turnover of WBC in humans. Further investigations are needed to better understand such effects and the exact molecular pathway involved.

The strengths and limitations of the study should also be acknowledged. Among strengths, is that we performed extensive chemical characterization of collected NPs, a component completely absent from most of the previous reports. This information might be useful to predict which component of these chemically complex NPs is responsible for the observed *in-vitro* effects. In addition, the *in-vitro* toxicity endpoints were chosen to match those measured in humans, enabling direct comparisons between the two systems, as illustrated in Table 4. The use of three cell types, representing different parts of the respiratory system is also informative. Among the limitations, high *in-vitro* doses (especially above 100 ug/mL) was already discussed. One important limitation is that we did not measure directly the endotoxin content of collected NP (in part due to insufficient NP material), even though we addressed possible endotoxin interferences by treating NPs with Polymyxin B. A more thorough characterization of the free radical generation of copier-emitted NP in acellular and cellular systems, and what components of the NP chemistry (organic fraction, water soluble metals, and nanoparticles, aged vs. fresh nanoaerosols), could not be completed due to insufficient NP mass, and should be studied further. It is also important to extend the *in-vitro* and *in vivo* evaluations to nanoparticles from other toner formulations (a.k.a. other photocopier brands) because their physicochemical and toxicological properties may differ.

## Conclusion

We show that nanoparticles in photocopy centers induced production of several inflammatory cytokines and chemokines in three human cell lines, as well as moderate apoptosis, but no DNA damage under *in-vitro* conditions. Up-regulation of inflammatory (TNF-α), apoptotic (CASP8 and p53), and oxidative stress genes (HO1) in THP-1 cells further substantiates these findings. Taken together, these *in-vitro* results are consistent with our previous human volunteers [17] and animal inhalation studies [39] and reinforce our earlier conclusion that copier-emitted nanoparticles are directly responsible for the induction of pro-inflammatory responses, likely through the oxidative stress pathway.

Further *in-vivo* and mechanistic studies are required to better understand trafficking patterns of these nanoparticles inside cells, molecular mechanisms involved, and to expand these investigations to nanoparticles originating from other toner manufacturers. More importantly, possible health effects of chronic exposures to copier-emitted nanoparticles on photocopier operators should be investigated.

## Abbreviations

NPs: Nanoparticles; IL: Interleukin; EGF: Epidermal growth factor; G-CSF: Granulocyte-colony stimulating factor; GM-CSF: Granulocyte macrophage- colony stimulating factor; MCP-1: Monocyte chemoattractant protein-1; TNF-α: Tumor necrosis factor α; VEGF: Vascular endothelial growth factor; VOCs: Volatile organic compounds.

## Competing interests

The authors declare they have no competing financial interests.

## Authors’ contributions

MK conceived and designed the study, performed cell culture and *in-vitro* assays, analyzed the results and drafted the manuscript. DB conceived, designed, and supervised the overall study, collected and characterized the nanoparticles, and participated in writing the manuscript. AP prepared and characterized nanoparticle dispersions for cell work and participated in writing relevant sections of the manuscript. SW helped with statistical analyses and participated in writing and editing the manuscript. TG helped with the study design, participated in writing and editing the paper. JC determined experimentally effective density of dispersions and performed the *in-vitro* dosimetry calculations, as well as participated in writing relevant sections of the manuscript. PD participated in the study design, airborne nanoparticle sample collection, conducted *in-vitro* to vivo dosimetry calculations, and participated in writing and editing of the manuscript. PG supervised the cell culture work and the *in-vitro* assays, as well as interpretation of cellular endpoints and participated in writing and editing the manuscript. JL conducted the gene expression bioassays, performed the corresponding gene expression data analysis, and helped with writing relevant sections of the manuscript. AG supervised the gene expression experiments, and participated in writing and editing the manuscript. All authors read and approved the final manuscript.
